# Excess Body Weight and the Risk of Second Primary Cancers Among Cancer Survivors

**DOI:** 10.1001/jamanetworkopen.2024.33132

**Published:** 2024-09-17

**Authors:** Clara Bodelon, Hyuna Sung, Ellen L. Mitchell, Emily L. Deubler, Christina C. Newton, Ahmedin Jemal, Lauren R. Teras, Alpa V. Patel

**Affiliations:** 1 Department of Population Science, American Cancer Society, Atlanta, Georgia; 2 Department of Surveillance & Health Equity Science, American Cancer Society, Atlanta, Georgia

## Abstract

**Importance:**

Little is known about the causes of second primary cancers among individuals with a history of cancer. Descriptive studies have suggested that lifestyle factors, including excess body weight, may be important.

**Objective:**

To investigate whether excess body weight is associated with the risk of a second primary malignant neoplasm among cancer survivors.

**Design, Setting, and Participants:**

This cohort study of adults in 21 states in the US used data from the Cancer Prevention Study II Nutrition cohort, a large prospective study that invited participants to respond to a survey in 1992 and biennial surveys starting in 1997, and who were followed-up through 2017. Eligible participants included those who received a diagnosis of a first primary nonmetastatic invasive cancer between 1992 and 2015. Data analysis occurred from September 2023 to March 2024.

**Exposure:**

Body mass index (BMI), computed from self-reported height and weight at the time of the first primary cancer diagnosis (mean [SD] years to diagnosis, 1.7 [1.5] years).

**Main Outcome and Measures:**

Main outcomes included a second primary cancer or an obesity-related second cancer. Cancer diagnoses were reported on biennial surveys and verified through medical record abstraction or linkage with state cancer registries.

**Results:**

This cohort included 26 894 participants who received a diagnosis of a first nonmetastatic primary cancer (mean [SD] age at first cancer diagnosis, 72.2 [6.5] years; 15 920 male [59.2%]). At the time of first diagnosis, 11 497 participants (42.8%) had overweight and 4684 (17.2%) had obesity. During a median (IQR) follow-up time of 7.9 (3.4-13.6) years, 3749 (13.9%) participants received a diagnosis of a second primary cancer, of which 1243 (33.2%) were obesity-related second primary cancers. Compared with cancer survivors whose BMI was in the normal range (18.5 to <25), there was 15% increased risk of any second primary cancer for those who had overweight (25 to <30; adjusted hazard ratio [aHR], 1.15; 95% CI, 1.07-1.25) and a 34% increased risk for those who had obesity (BMI ≥30; aHR, 1.34; 95% CI, 1.21-1.48), with greater risk for obesity-related second primary cancers, including a 40% increased risk for those with overweight (aHR, 1.40; 95% CI, 1.22,-1.61) and a 78% increased risk for those with obesity (aHR, 1.78; 95% CI, 1.51-2.11).

**Conclusions and Relevance:**

In this cohort study of older survivors of nonmetastatic cancer, those who had overweight or obesity at the time of their first cancer diagnosis were at higher risk of developing a second cancer, especially an obesity-related second cancer. Given the high prevalence of overweight and obesity among cancer survivors, it is important to promote survivorship care guidelines recommending weight management and increase awareness of second cancers among physicians and cancer survivors.

## Key Points

**Question:**

Is excess body weight associated with an increased risk of second cancers among cancer survivors?

**Findings:**

In this cohort of 26 894 older survivors of nonmetastatic cancer, 42.8% had overweight and 17.2% had obesity at their first primary cancer diagnosis. Having obesity was associated with a 34% increased risk of developing a second cancer, and a 78% increased risk of developing an obesity-related second cancer.

**Meaning:**

These results suggest that excess body weight increases the risk of second cancers among cancer survivors and emphasize the importance of recommending healthy weight guidelines and heightening awareness of second cancers among physicians of cancer survivors.

## Teaser

This cohort study analyzes associations of excess body weight with the risk of developing a second primary cancer among survivors of a first primary cancer.

## Introduction

The population of cancer survivors has increased substantially in recent decades due to advances in early detection, improved cancer treatments, and growth of the population.^1^ However, cancer survivors face many medical challenges,^2^ one of which is a higher risk of developing another primary cancer compared with the expected risk in the general population of similar age.^3,4^ It is estimated that subsequent malignant neoplasms in survivors of adult cancers constitute approximately 20% of all new cancer diagnoses each year,^5^ and they are the leading cause of morbidity and mortality among cancer survivors. Thus, understanding the causes of second primary cancers could have important public health and clinical implications.

Prior studies examining the causes of second cancers among survivors of childhood cancers have found that treatment and genetics play major roles.^5^ However, little is known about the causes of second primary cancers among survivors of adult-onset cancers. It is estimated that less than 10% of second primary cancers occurring among adults can be attributed to radiotherapy.^6^ While other treatments have been associated with second cancers,^7^ it is unknown how many second adult-onset cancers can be attributed to these treatments. However, the importance of lifestyle factors, environmental exposures, and genetic susceptibility in the causes of second cancers among adult cancer survivors has been suggested.^3,8,9,10,11^ A recent analysis^10^ of cancer registry data found that individuals with a history of obesity-related cancers are at an elevated risk of developing and dying from subsequent obesity-related cancers. However, registry-based studies lack information on important risk factors and confounder factors^12^ and assume group-level associations as proxies of individual-level associations, which may result in biased estimates. Therefore, the conclusions based on ecological observations are merely hypothesis generating. To our knowledge, the association of obesity with the risk of second primary cancers has not been comprehensively examined in a prospective cohort study. Cohort studies do not have the limitations of registry-based studies and are less likely to have other sources of biases such as recall bias or reverse causation. Prior studies have focused only on survivors of a specific cancer site (eg, female breast and colorectum).^13,14^

Herein, we examine the association of body mass index (BMI) and the incidence of second primary cancers among a large cohort of adults who received a diagnosis of a first primary cancer using longitudinal lifestyle factor data, long-term follow-up, and systematic collection of cancer diagnoses.

## Methods

### Study Population

This Cancer Prevention Study II (CPS-II) Nutrition Cohort study was approved by the Emory University institutional review board. This study was conducted in accordance with the Strengthening the Reporting of Observational Studies in Epidemiology (STROBE) reporting guidelines for cohort studies. The CPS-II Nutrition Cohort is a prospective cohort of more than 184 000 participants who were part of the CPS-II Mortality Cohort (established in 1982) and were invited to participate in a survey in 1992 to update demographic information, lifestyle factors, and followed-up for cancer incidence.^15^ Cohort members provide written informed consent for medical records release. At the time of each mailed questionnaire, cohort members were informed that their identifying information was used to link with cancer registries and death indexes. Participants enrolled in the CPS-II Nutrition Cohort received biennial questionnaires starting in 1997. Response rates to the biennial questionnaires range from 78.1% to 92.1%. Cancer diagnoses were reported on biennial CPS-II Nutrition Cohort surveys and verified through medical record abstraction or linkage with state cancer registries. The validity of this method has been shown to have a sensitivity of 93% in the previously reported CPS-II Nutrition Cohort.^16^ Information on clinical characteristics was captured from medical records to match data cancer registries. Cancer types were classified per the *International Classification of Diseases for Oncology, Third Edition* (*ICD-O-3*)^17^ and deaths were identified through linkage with the National Death Index. Participants were considered lost to follow-up if (1) they were alive but did not return any follow-up questionnaires or (2) they died and did not return the biennial questionnaire prior to their death date. They were censored at the date of their last returned questionnaire. Participants who died but returned the biennial questionnaire prior to their death date were censored at their death date. The eFigure in Supplement 1 provides an overview of inclusion of participants in the analysis, with criteria for exclusion, and reasons for censoring. Specifically, we identified individuals in the CPS-II Nutrition Cohort who developed a first verified primary invasive cancer (excluding nonmelanoma skin cancers) between January 1, 1992, and June 30, 2015 (36 529 individuals), and were followed-up to June 30, 2017, for a second primary cancer diagnosis. We then excluded participants who had a diagnosis of in situ or distant-stage disease (6357 individuals) or were 85 years or older at the time of their first primary diagnosis (1463 individuals). We further excluded participants who had a second primary cancer diagnosis within 60 days of their first primary diagnosis (333 individuals) or died within 60 days of their first cancer diagnosis or were lost to follow-up (837 individuals). Follow-up through 2017 was completed for 88.4% of person-years (eFigure in Supplement 1).

### Assessment of BMI, Demographic and Lifestyle Factors, and Treatment Information

BMI (calculated as weight in kilograms divided by height in meters squared), and other demographic and lifestyle factors were self-reported and obtained from the surveys. BMI was computed from data obtained from the closest survey before the date of the first primary diagnosis. Participants who had underweight (BMI <18.5) at the time of the first primary cancer were excluded (645 individuals). BMI was also assessed at 3 intervals after the diagnosis of the first primary cancer: up to 2 years postdiagnosis, between 2 and 5 years postdiagnosis, and more than 5 years postdiagnosis. If multiple surveys to assess BMI were available in any of the intervals, the survey with the closest measure to the first primary diagnosis was used.

First course treatment for the first primary cancer was available from linkage to Medicare claims data when received within 6 months of the cancer diagnosis (available for cases diagnosed from 1999 to 2017) and supplemented with self-reported treatment for survivors of breast, colorectal, and prostate cancers. Medicare claims for cancer treatment (chemotherapy and radiation therapy) were identified using established methods and updated with Healthcare Common Procedure Coding System, *International Classification of Diseases, Ninth Revision (ICD-9)*, and *International Statistical Classification of Diseases, Tenth Revision, Clinical Modification (ICD-10-CM)*.^18^ Among Medicare-eligible cancer survivors (11 528 individuals) chemotherapy was missing for 60 survivors (0.5%) and radiation therapy was missing for 51 survivors (0.4%). For participants who had treatment information available from both sources (Medicare claims and self-report; 5698 participants for chemotherapy and 5715 participants for radiation therapy), the percentage agreement between the 2 sources was 96.5% for chemotherapy and 89.0% for radiation therapy. Treatment was categorized as surgery (yes, no, or unknown; only from self-report), chemotherapy (yes, no, or unknown) and radiotherapy (yes, no, or unknown).

### Ascertainment of Second Primary Cancers and Outcomes of Interest

Second primary malignant neoplasms were ascertained following the Surveillance, Epidemiology and End Results Program 2007 Multiple Primary and Histology Coding Rules.^19^ We restricted second malignant neoplasms to any new verified primary diagnosis, excluding nonmelanoma skin cancers, occurring at a different site from the first primary cancer to avoid misclassification of a local recurrence as a second primary cancer. Cancers occurring in paired organs were considered occurring at the same site as the first primary cancer and were not included as second primary cancers.

Outcomes of interest included any second primary cancer and obesity-related second cancers. Cancers were considered obesity-related if they were 1 of the 14 cancers described by the International Agency for Research on Cancer working group as being causally related to excess body fatness.^20^ They included the following: esophageal adenocarcinoma, gastric cardia, colon, rectum, liver, gallbladder, pancreas, postmenopausal breast cancer, corpus uteri, ovary, kidney, meningioma, thyroid, and multiple myeloma (eTable 1 in Supplement 1). We also explore the risk of obesity-related specific second cancers to cancer sites with at least 100 cases (breast, colorectum, kidney, and pancreas).

### Statistical Analysis

Cancer survivors were followed up starting 60 days after the diagnosis of the first primary cancer until the diagnosis of a second primary cancer, death, loss to follow-up, or June 30, 2017, whichever came first. Any cause of death was a competing event for the incidence of a second primary cancers and its occurrence became a censoring time point in the analysis of second cancers. To describe the incidence of second cancers by BMI categories (18.5 to <25.0, 25.0 to <30.0, and BMI ≥30.0) in the presence of death as a competing event the cumulative incidence function was used.^21^ It was computed using the cuminc() function from the cmprsk package for R statistical software (R Project for Statistical Computing), which implements the method described by Fine et al.^22^ It estimates specific subdistribution hazard functions for each event: second primary cancers and death.^21,23^ The Gray test, a modified χ^2^ test that compares the subdistribution hazard functions, was used to compare cumulative incidence across groups.^23^ For analyses of the incidence of obesity-related second cancers, the methods used were the same, but competing risks were any cause of death or non–obesity-related second cancers. Cox proportional hazard regression models were used to calculate hazard ratios (HRs) and 95% CIs for the risk of developing a second cancer associated with BMI. They were adjusted for race and ethnicity (non-Hispanic White and other races or ethnicities) and smoking (never, former, current, or missing) and the baseline hazard function stratified by age at first cancer diagnosis (every 1 year), sex (male and female), year of first cancer diagnosis, and stage (localized, regional, or unknown) to account for significant departures of the proportional hazard assumption based on tests of the slopes of the Schoenfeld residuals.^24^ BMI did not violate the proportional hazard assumption based on the same test. Race and ethnicity were self-reported in the 1982 questionnaire with the following response categories: White, Black, Hispanic, Oriental, and other (defined as any race or ethnicity not otherwise specified). Participants were able to select multiple categories; race and ethnicity were included to describe the study population. Smoking status was obtained from the closest survey before the date of the first primary diagnosis. Physical activity was ascertained in the 1999, 2001, 2005, 2009, and 2011 surveys. We restricted analyses adjusted for physical activity to survivors with prediagnosis BMI ascertained from the previous surveys who had nonmissing physical activity data in that survey. To assess the long-term association of obesity with the risk of second primary cancers, associations were also estimated among 1- and 5-year cancer survivors.

For the analyses investigating the risk of specific second cancers, the same models as the ones previously described were run. The cohort of survivors for the risk of postmenopausal breast cancer was restricted to postmenopausal females who did not receive a first breast cancer diagnosis, because these individuals were not at risk of developing a second breast cancer by design. Similarly, the cohort of survivors for the risk of the other specific second cancers (colorectum, kidney, and pancreas) did not include survivors with a first primary diagnosis of the same type.

Sensitivity analyses were conducted where models were also adjusted for first course treatment and comorbidities at the time of the first cancer diagnosis. To examine whether the associations differed by participants characteristics, subgroups analyses were conducted by age (<70 and ≥70 years) and sex, as well as restricted to nonsmokers. We also conducted analysis restricted to Medicare-eligible participants for whom we had treatment information from Medicare claims data. Additional analyses were conducted in BMI-related first cancers and after excluding breast and colorectal cancer survivors because associations for survivors of these cancers have been previously reported.^13,14^ Finally, we estimated subdistribution HRs with Fine and Gray regression models to estimate the risk of second cancers in the presence of competing events.^21^

Analyses were conducted using the R software version 4.3.1 or SAS Studio version 3.82 (SAS Institute). All statistical tests were 2-sided, and *P* < .05 was considered statistically significant. Analysis occurred from September 2023 to March 2024.

## Results

This cohort included 26 894 participants who received a diagnosis of a first nonmetastatic primary cancer (mean [SD] age at first cancer diagnosis, 72.2 [6.5] years; 23 023 [85.6%] 65 years or older; 15 920 male [59.2%]; 108 Asian [0.4%]; 344 Black [1.3%]; 26 257 White [97.6%]) (Table 1). Survivors were more likely to be never smokers (10 572 participants [39.3%]) or former smokers (14 414 participants [53.6%]) and be diagnosed at localized stage (19 142 individuals [71.2%]). These characteristics were similar across BMI categories. The most common first primary cancers were prostate (9411 individuals [35.0%]), breast (5145 individuals [19.1%]) and colorectum (2556 individuals [9.5%]). Of all first primary cancers, 10 660 (39.6%) were obesity related.

**Table 1.** Characteristics of Participants in the Cancer Prevention Study II Who Received a Diagnosis of a First Nonmetastatic Primary Cancer, Overall and by BMI Categories

Characteristics	Survivors, No. (%)			
	All survivors (N = 26 894)	Survivors by BMI categories^a^		
		18.5 to <25.0 (n = 10 713)	25.0 to <30.0 (n = 11 497)	≥30.0 (n = 4684)
Age at first diagnosis, mean (SD), y	72.2 (6.5)	72.6 (6.7)	72.1 (6.3)	71.3 (6.3)
Age at first diagnosis, y				
<65	3871 (14.4)	1503 (14.0)	1599 (13.9)	769 (16.4)
65 to <70	5971 (22.2)	2193 (20.5)	2654 (23.1)	1124 (24.0)
70 to <75	7401 (27.5)	2771 (25.9)	3259 (28.3)	1371 (29.3)
75 to <80	6419 (23.9)	2687 (25.1)	2712 (23.6)	1020 (21.8)
80 to <85	3232 (12.0)	1559 (14.6)	1273 (11.1)	400 (8.5)
Sex				
Female	10 974 (40.8)	5173 (48.3)	3569 (31.0)	2232 (47.7)
Male	15 920 (59.2)	5540 (51.7)	7928 (69.0)	2452 (52.3)
Race or ethnicity				
Asian	108 (0.4)	57 (0.5)	44 (0.4)	7 (0.2)
Black	344 (1.3)	85 (0.8)	156 (1.4)	103 (2.2)
Hispanic	78 (0.3)	32 (0.3)	36 (0.3)	10 (0.2)
White	26 257 (97.6)	10 492 (97.9)	11 228 (97.7)	4537 (96.9)
Other^b^	34 (0.1)	14 (0.1)	11 (0.1)	9 (0.2)
Missing	73 (0.3)	33 (0.3)	22 (0.2)	18 (0.4)
Year of first diagnosis				
1992-1995	4330 (16.1)	1791 (16.7)	1864 (16.2)	675 (14.4)
1996-2000	8661 (32.2)	3545 (33.1)	3713 (32.3)	1403 (30.0)
2001-2005	7030 (26.1)	2662 (24.8)	3108 (27.0)	1260 (26.9)
2006-2010	4763 (17.7)	1893 (17.7)	1967 (17.1)	903 (19.3)
2011-2015	2110 (7.8)	822 (7.7)	845 (7.3)	443 (9.5)
Education level				
Some high school or less	1580 (5.8)	454 (4.3)	740 (6.5)	386 (8.3)
High school graduate	6137 (22.8)	2173 (20.3)	2635 (22.9)	1329 (28.4)
Vocational or trade school	1577 (5.9)	559 (5.2)	707 (6.1)	311 (6.6)
Some college	6025 (22.4)	2442 (22.8)	2504 (21.8)	1079 (23.0)
College graduate	5851 (21.8)	2581 (24.1)	2482 (21.6)	788 (16.8)
Graduate school	5563 (20.7)	2441 (22.8)	2364 (20.6)	758 (16.2)
Missing or unknown	161 (0.6)	63 (0.6)	65 (0.6)	33 (0.7)
Insurance^c^				
Employer insurance	6894 (26.0)	2605 (24.3)	2994 (26.0)	1385 (29.6)
Self-bought insurance	1374 (5.1)	558 (5.2)	531 (4.6)	285 (6.1)
Medicaid or Medicare	13 412 (49.9)	5643 (52.7)	5739 (49.9)	2030 (43.3)
Military insurance	231 (0.9)	90 (0.8)	98 (0.9)	43 (0.9)
Other insurance	459 (1.7)	171 (1.6)	193 (1.7)	95 (2.0)
No insurance	76 (0.3)	30 (0.3)	22 (0.2)	24 (0.5)
Missing	4358 (16.2)	1616 (15.1)	1920 (16.7)	822 (17.5)
Smoking status^d^				
Never	10 572 (39.3)	4427 (41.3)	4293 (37.3)	1852 (39.5)
Former	14 414 (53.6)	5325 (49.7)	6503 (56.6)	2586 (55.2)
Current	1825 (6.8)	928 (8.7)	664 (5.8)	233 (5.0)
Missing	83 (0.3)	33 (0.3)	37 (0.3)	13 (0.3)
Alcohol consumption^d^				
Nondrinker	9484 (35.3)	3492 (32.6)	3960 (34.4)	2032 (43.4)
<1 Drink weekly	2557 (9.5)	979 (9.1)	1089 (9.5)	489 (10.4)
1-6 Drinks weekly	6621 (24.6)	2877 (26.9)	2838 (24.7)	906 (19.3)
1 Drink daily	2859 (10.6)	1329 (12.4)	1229 (10.7)	301 (6.4)
≥2 Drinks daily	2247 (8.4)	923 (8.6)	1028 (8.9)	296 (6.3)
Unknown or missing	3126 (11.6)	1113 (10.4)	1353 (11.8)	660 (14.1)
Menopausal status (females only)^d^				
Premenopausal	71 (0.7)	44 (0.9)	11 (0.3)	16 (0.7)
Postmenopausal	10 903 (99.3)	5129 (99.1)	3558 (99.7)	2216 (99.3)
Diabetes^d^				
No	22 997 (85.5)	9646 (90.0)	9811 (85.3)	3540 (75.6)
Yes	3897 (14.5)	1067 (10.0)	1686 (14.7)	1144 (24.4)
Hypertension^d^				
No	13 870 (51.6)	6628 (61.9)	5599 (48.7)	1643 (35.1)
Yes	13 024 (48.4)	4085 (38.1)	5898 (51.3)	3041 (64.9)
Cardiovascular disease^d^^,^^e^				
No	21 342 (79.4)	8741 (81.6)	8955 (77.9)	3646 (77.8)
Yes	5552 (20.6)	1972 (18.4)	2542 (22.1)	1038 (22.2)
Stage of first cancer				
Localized	19 142 (71.2)	7571 (70.7)	8307 (72.3)	3264 (69.7)
Regional^f^	6319 (23.5)	2551 (23.8)	2577 (22.5)	1191 (25.4)
Unknown	1433 (5.3)	591 (5.5)	613 (5.3)	229 (4.9)
Surgery for first cancer^g^				
No	4574 (17.0)	1699 (15.9)	2229 (19.4)	646 (13.8)
Yes	9105 (33.9)	3772 (35.2)	3749 (32.6)	1584 (33.8)
Missing	13 215 (49.1)	5242 (48.9)	5519 (48.0)	2454 (52.4)
Chemotherapy for first cancer^g^				
No	15 842 (58.9)	6303 (58.8)	6923 (60.2)	2616 (55.8)
Yes	3786 (14.1)	1523 (14.2)	1551 (13.5)	712 (15.2)
Missing	7266 (27.0)	2887 (26.9)	3023 (26.3)	1356 (28.9)
Radiation therapy for first cancer^g^				
No	10 892 (40.5)	4332 (40.4)	4705 (40.9)	1855 (39.6)
Yes	8746 (32.5)	3498 (32.7)	3775 (32.8)	1473 (31.4)
Missing	7256 (27.0)	2883 (26.9)	3017 (26.2)	1356 (28.9)
Most common first cancers^h^				
Bladder	750 (2.8)	282 (2.6)	350 (3.0)	118 (2.5)
Breast	5145 (19.1)	2386 (22.3)	1720 (15.0)	1039 (22.2)
Colorectum	2556 (9.5)	1004 (9.4)	1049 (9.1)	503 (10.7)
Lung	1659 (6.2)	722 (6.7)	687 (6.0)	250 (5.3)
Melanoma	1395 (5.2)	580 (5.4)	594 (5.2)	221 (4.7)
Non-Hodgkin Lymphoma	1191 (4.4)	480 (4.5)	494 (4.3)	217 (4.6)
Prostate	9411 (35.0)	3359 (31.4)	4722 (41.1)	1330 (28.4)
Endometrial	993 (3.7)	375 (3.5)	306 (2.7)	312 (6.7)

Abbreviation: BMI, body mass index.

^a^ BMI was calculated as weight in kilograms divided by height in meters squared.

^b^ Other was defined as any race or ethnicity not otherwise specified.

^c^ Self-report from the 1997 questionnaire.

^d^ At the time of the first primary diagnosis.

^e^ Cardiovascular disease was defined as having any self-report episode of heart disease or stroke.

^f^ Includes 425 cases of blood cancers that were coded as distant.

^g^ First course treatment from linkage to Medicare claims data (available for cases diagnosed from 1999-2017) and supplemented with self-reported treatment for survivors of breast, colorectal, and prostate cancers.

^h^ Only shown those with frequencies greater than 2.5%.

**Figure 1.**
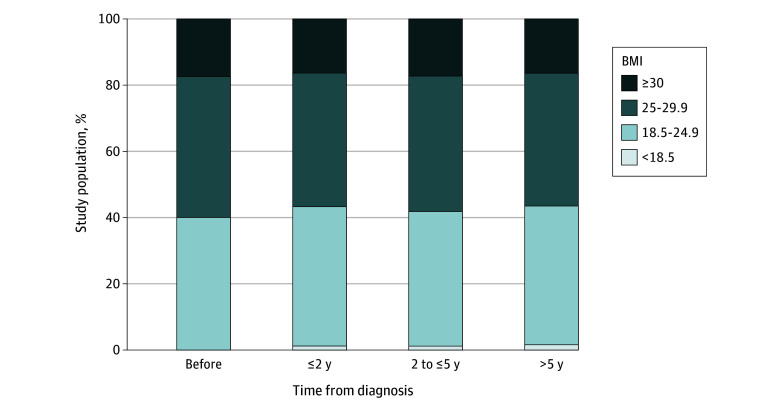
Body Mass Index (BMI) Among Survivors of a First Primary Cancer Percentage of the study population in each of the 4 categories of BMI (calculated as weight in kilograms divided by height in meters squared) was assessed at different time intervals with respect to the diagnosis of their first primary cancer. Time intervals were prior to the first primary cancer diagnosis (mean [SD] time, 1.7 [1.5] years), up to 2 years postdiagnosis (mean [SD] time, 0.9 [0.6] years), between 2 and 5 years postdiagnosis (mean [SD] time, 3.1 [0.7] years), and more than 5 years postdiagnosis (mean [SD] time, 6.1 [0.8] years). Participants who had a prediagnosis BMI less than 18.5 were excluded from the analysis.

BMI was assessed prior to the first primary cancer diagnosis (mean [SD] years from BMI assessment to diagnosis, 1.7 [1.5] years), up to 2 years postdiagnosis (mean [SD] time, 0.9 [0.6] years), between 2 and 5 years postdiagnosis (mean [SD] time, 3.1 [0.7] years), and more than 5 years postdiagnosis (mean [SD] time, 6.1 [0.8] years) (Figure 1). At the time of first diagnosis, 11 497 participants (42.8%) had overweight and 4684 (17.2%) had obesity, and similar percentages were observed during the follow-up period (Table 1 and Figure 1). Based on this observation, prediagnostic BMI was used as our main exposure in the analyses below.

**Figure 2.**
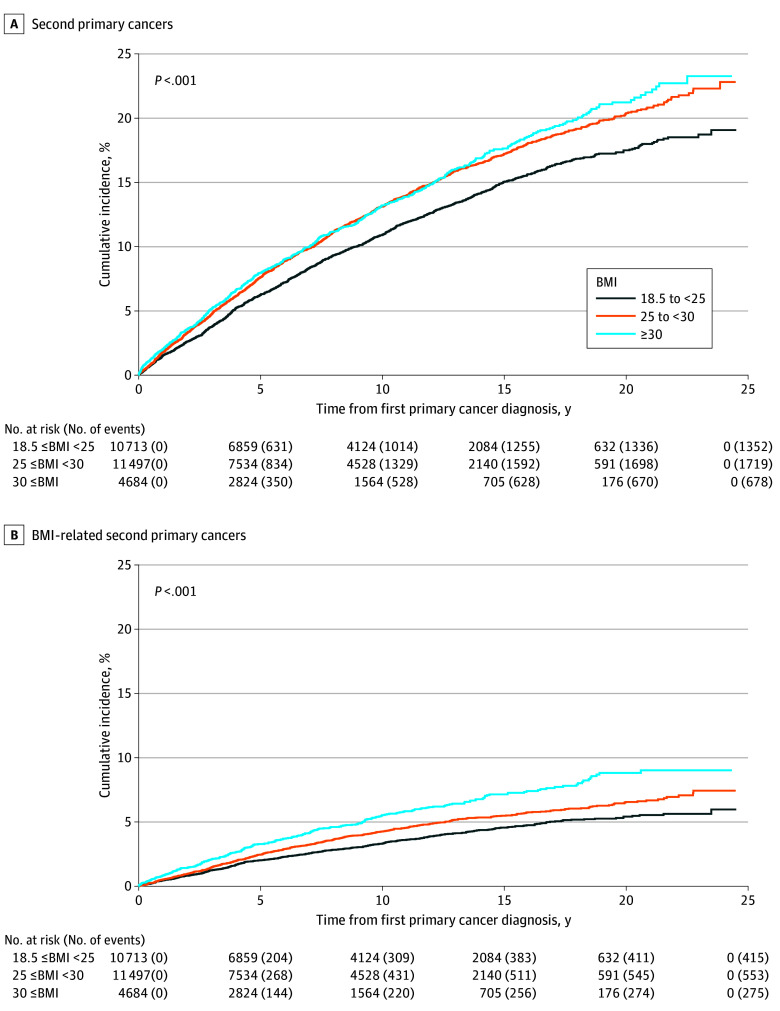
Cumulative Incidence Curves Cumulative incidence of any second primary cancers (A) and body mass index (BMI)–related second primary cancers (B) by prediagnostic BMI categories among 26 894 individuals who received a diagnosis of a first primary cancer between 1992 and 2015 and followed-up through 2017. BMI was calculated as weight in kilograms divided by height in meters squared.

During a median (IQR) follow-up of 7.9 (3.4-13.6) years, 3749 participants (13.9%) received a diagnosis of a second cancer, of which 1243 (33.2%) were obesity-related second cancers. Approximately 90% of second malignant neoplasms (3356 [89.5%]) were diagnosed at least 1 year after date of diagnosis of the first cancer, and more than 50% of them (1979 [52.8%]) were diagnosed at least 5 years after the diagnosis of the first cancer. The mean (SD) age at the second cancer diagnosis was 77.5 (6.3) years (eTable 2 in Supplement 1). Higher BMI was associated with greater incidence of second cancers among cancer survivors (Figure 2). Among survivors who had obesity (BMI ≥30.0), the 10-year cumulative incidence was 13.2% (95% CI, 12.2%-14.3%) for any second primary cancers and 5.5% (95% CI, 4.8%-6.3%) for obesity-related second cancers.

Every 5-kg/m^2^ increase in BMI was associated with a 13% increased risk of a second cancer (adjusted HR [aHR], 1.13; 95% CI, 1.08-1.18), with the magnitudes of the associations similar for 1- and 5-year survivors (Table 2). The risk of developing an obesity-related second cancer was considerably greater, with a 28% increased risk for every 5-kg/m^2^ increase in BMI (aHR, 1.28; 95% CI, 1.20-1.37). Compared with cancer survivors whose BMI was in the normal range (18.5≤ to <25.0), there was a 15% increased risk of any second cancer for those who had overweight (BMI, 25.0 to <30.0; aHR, 1.15; 95% CI, 1.07-1.25) and a 34% increased risk for those with obesity (BMI, ≥30.0; aHR, 1.34; 95% CI, 1.21-1.48); there was also a 40% increased risk of obesity-related cancers for those who had overweight (aHR, 1.40; 95% CI, 1.22-1.61) and a 78% increased risk for those with obesity (aHR, 1.78; 95% CI, 1.51-2.11). Risk of second cancers was similar when BMI was ascertained at least 2 years prior to the first cancer diagnosis (eTable 3 in Supplement 1). Associations were also similar when models were also adjusted for first course of treatment for the first primary cancer and comorbidities, including diabetes, hypertension, and prevalent cardiovascular disease (eTable 4 in Supplement 1).

**Table 2.** BMI and the Risk of Developing Second Primary Cancers Among Participants in the Cancer Prevention Study II Who Received a Diagnosis of a First Primary Cancer

BMI variable by outcome^a^	All survivors (N = 26 894)		1-y Survivors (n = 24 580)		5-y Survivors (n = 17 473)	
	Second cancers, No.	aHR (95% CI)^b^	Second cancers, No.	aHR (95% CI)^b^	Second cancers, No.	aHR (95% CI)^b^
All second cancers						
Continuous (per 5 kg/m^2^)	3749	1.13 (1.08-1.18)	3356	1.12 (1.07-1.17)	1979	1.11 (1.04-1.17)
Categories						
18.5-24.9	1352	1.00 [Reference]	1217	1.00 [Reference]	736	1.00 [Reference]
25.0-29.9	1719	1.15 (1.07-1.25)	1546	1.15 (1.06-1.25)	908	1.12 (1.00-1.24)
≥30.0	678	1.34 (1.21-1.48)	593	1.30 (1.17-1.45)	335	1.28 (1.11-1.47)
BMI-related second cancers						
Continuous (per 5 kg/m^2^)	1243	1.28 (1.20-1.37)	1117	1.27 (1.18-1.37)	638	1.27 (1.15-1.40)
Categories						
18.5-24.9	415	1.00 [Reference]	373	1.00 [Reference]	214	1.00 [Reference]
25.0-29.9	553	1.40 (1.22-1.61)	502	1.41 (1.22-1.64)	292	1.42 (1.17-1.73)
≥30.0	275	1.78 (1.51-2.11)	242	1.72 (1.44-2.06)	132	1.67 (1.32-2.13)

Abbreviations: aHR, adjusted hazard ratio; BMI, body mass index.

^a^ BMI was calculated as weight in kilograms divided by height in meters squared.

^b^ Adjusted for race, smoking, and baseline hazard stratified by age, sex, year of diagnosis, and stage.

Higher BMI was associated with significant increased risks of second colorectal cancer, one of the most common obesity-related second cancers (Table 3). Every 5-kg/m^2^ increase in BMI had a 13% increased risk of a second breast cancer (aHR, 1.13; 95% CI, 0.98-1.31), but the finding was not statistically significant; every 5-kg/m^2^ increase was associated with a 42% increased risk of a second colorectal cancer (aHR, 1.42; 95% CI, 1.26-1.61). Every 5-kg/m^2^ increase in BMI was associated with a 70% increased risk of a second kidney cancer among all survivors (aHR, 1.70; 95% CI, 1.35-2.15), but the association was attenuated for 5-year survivors.

**Table 3.** BMI and the Risk of Developing Specific BMI-Associated Second Cancers Among Participants in the Cancer Prevention Study II Who Received a Diagnosis of a First Primary Cancer^a^

BMI variable by outcome^b^	All survivors		1-y Survivors		5-y Survivors	
	Second cancers, No.	aHR (95% CI)^c^	Second cancers, No.	aHR (95% CI)^c^	Second cancers, No.	aHR (95% CI)^c^
Postmenopausal breast cancer (females only)^d^						
Continuous (per 5 kg/m^2^)	243	1.13 (0.98-1.31)	224	1.13 (0.97-1.32)	112	1.19 (0.96-1.48)
Categories						
18.5-24.9	100	1.00 [Reference]	91	1.00 [Reference]	43	1.00 [Reference]
25.0-29.9	86	1.21 (0.84-1.74)	83	1.31 (0.90-1.92)	46	1.54 (0.90-2.62)
≥ 30.0	57	1.32 (0.89-1.96)	50	1.30 (0.85-1.98)	23	1.33 (0.71-2.47)
Colorectal cancer^e^						
Continuous (per 5 kg/m^2^)	400	1.42 (1.26-1.61)	361	1.39 (1.22-1.59)	197	1.50 (1.25-1.80)
Categories						
18.5-24.9	109	1.00 [Reference]	100	1.00 [Reference]	53	1.00 [Reference]
25-29.9	214	1.88 (1.46-2.42)	193	1.83 (1.40-2.38)	104	1.84 (1.27-2.65)
≥30	77	2.05 (1.49-2.82)	68	1.93 (1.37-2.70)	40	2.31 (1.47-3.63)
Kidney cancer^f^						
Continuous (per 5 kg/m^2^)	117	1.70 (1.35-2.15)	97	1.57 (1.20-2.05)	54	1.18 (0.80-1.74)
Categories						
18.5-24.9	25	1.00 [Reference]	23	1.00 [Reference]	15	1.00 [Reference]
25-29.9	57	1.71 (1.03-2.83)	48	1.58 (0.92-2.71)	30	1.49 (0.75-2.96)
≥30	35	3.50 (2.01-6.10)	26	2.67 (1.45-4.91)	9	1.39 (0.58-3.38)
Pancreatic cancer^g^						
Continuous (per 5 kg/m^2^)	138	1.08 (0.86-1.36)	126	1.06 (0.83-1.36)	86	1.04 (0.78-1.40)
Categories						
18.5-24.9	57	1.00 [Reference]	51	1.00 [Reference]	35	1.00 [Reference]
25-29.9	57	0.90 (0.60-1.37)	53	0.94 (0.61-1.45)	37	0.98 (0.58-1.65)
≥30	24	1.05 (0.61-1.82)	22	1.06 (0.60-1.89)	14	0.90 (0.44-1.83)

Abbreviations: aHR, adjusted hazard ratio; BMI, body mass index.

^a^ Analyses were restricted to cancer sites with at least 100 second cancers.

^b^ BMI was calculated as weight in kilograms divided by height in meters squared.

^c^ Adjusted for race, smoking, and baseline hazard stratified by age, sex, year of diagnosis, and stage.

^d^ The number of female cancers survivors at risk of developing a second primary postmenopausal breast cancer included 5833 individuals overall, 5057 1-year survivors, and 3152 5-year survivors.

^e^ The number of cancer survivors at risk of developing a second primary colorectal cancer included 24 338 individuals overall, 22 198 1-year survivors, and 15 866 5-year survivors.

^f^ The number of cancer survivors at risk of developing a second primary pancreatic cancer included 26 344 individuals overall, 24 083 1-year survivors, and 17 161 5-year survivors.

^g^ The number of cancer survivors at risk of developing a second primary kidney cancer included 26 568 individuals overall, 24 426 1-year survivors, and 17 450 5-year survivors.

In subgroup analyses, associations were similar when stratified by age and sex (eTable 5 and eTable 6 in Supplement 1). When we limited the analysis to nonsmokers or survivors with physical activity information, our effect size estimates were similar, albeit statistically nonsignificant in some subgroups likely due to the smaller sample sizes (eTable 7 and eTable 8 in Supplement 1). Sensitivity analysis among survivors who were eligible for Medicare with more comprehensive treatment data were also conducted, and results did not differ from the main results (eTable 9 and eTable 10 in Supplement 1). Survivors of a first obesity-related cancer were at significant increased risk of developing a BMI-related second cancer (eTable 11 in Supplement 1). Analyses were also run among survivors of a first cancer that was not breast or colorectal cancer, and results were unchanged (eTable 12 in Supplement 1). Finally, given the older population of survivors in our analysis, with death being an important competing risk which could affect risk estimates and inference, we conducted Fine and Gray regression models accounting for competing risks (eTable 13 in Supplement 1). Our results were qualitatively the same.

## Discussion

In this large cohort study of predominantly older survivors of nonmetastatic cancer, we found that those who had overweight or obesity at the time of the first diagnosis were at an increased risk of any second cancer and obesity-related cancers. These findings support the hypothesis that excess body weight increases the risk of second primary cancers, particularly for obesity-related cancer. Our results extend the body of literature of adverse outcomes associated with excess body weight among cancer survivors.^25^ Excess body weight is a modifiable risk factor, and weight loss to reach a healthy body weight among cancer survivors may result in substantial health and quality of life benefits. Older survivors may face unique challenges to achieve dietary or physical activity goals needed to reach or maintain a healthy weight, due to physical, physiological, and metabolic changes.^26^ A personalized weight management plan, which may include a nutrition plan, and a physical activity regimen that is shared with an interdisciplinary care team to ensure adequate social support and additional resources may result in long-term healthy behaviors.^27,28,29^

Previous results found that increased BMI was associated with an increased risk of second cancers among breast and colorectal cancer survivors.^13,14^ We extended these findings to all cancer survivors and found that increased BMI was associated with an increased risk of second cancers among all survivors. Moreover, a significant increased risk was observed even after excluding breast and colorectal cancer survivors, suggesting that the findings are generalizable to cancer survivors and not driven by associations in breast and colorectal cancer survivors alone.

To our knowledge, our study is the first to look at the associations of BMI with second cancers in older cancer survivors. The mean age of the cancer survivors in our analysis was approximately 10 years older than some of those previously published in adult second cancers looking at the associations of BMI with the risk of subsequent cancers.^13,14^ Despite more frequent diagnoses of second primary cancers among older cancer survivors, research examining the risk factors of second primary cancers among older cancer survivors has been sparse. Some of the reasons for the lack of studies in this older population is the result of underreporting of second malignant neoplasms in older patients.^30^ Other reasons may be that they have more competing causes of mortality, generally fewer remaining years to develop a second cancer, or receiving a diagnosis of indolent cancers.^4^ It is important to continue to study the older population of survivors, who have unique challenges due to competing health-related and aging-related conditions which could influence their risk of second cancers compared with younger survivors.^31,32^ Moreover, older cancer survivors have been underrepresented in research,^33^ and studies in this population have been identified as a priority area.^32,34^

### Strengths and Limitations

Strengths of this study include the large sample size of the cohort with its prospective nature as well as the number of second cancers, the systematic collection of risk factor data with high response rates and cancer diagnoses, and long-term follow-up. For studies of the causes of second cancers, the time of exposure ascertainment close to the first primary diagnosis and the availability of follow-up questionnaires after the initial diagnosis are key to minimizing misclassification of the exposure within cohort participants. This has been a limitation of previous cohort efforts for second cancers.^35^ With the availability of biennial questionnaires during the follow-up period, we were able to identify variables close to the initial diagnosis, when the timing of the exposure is closely aligned with a causal framework. Furthermore, the high frequency of questionnaires also minimizes the selection of individuals who need to survive long enough to complete the follow-up questionnaires. Another strength of the study was the exclusion of survivors with metastatic disease, which may have disease-related weight loss. We also tried to minimize the potential for reverse causation by looking at BMI and the risk of second cancers among all survivors, 1-year survivors, and 5-year survivors, with similar associations in these groups of survivors.

Despite these advantages, our study also has limitations. Multiple primary cancers in the same site were not recorded to avoid misclassification of new primary cancers with recurrences or metastatic disease. Given that multiple primary cancers can occur in the same site as the first primary cancer,^36^ our results likely underestimated the magnitude of the association of excess body weight with the risk of second primary cancers. We used BMI as a measure of excess body fat. While BMI is easy to collect in epidemiologic studies, it is an imperfect measure of body composition, unable to differentiate between fat and lean mass or to characterize the distribution of adipose tissue in the body. Although cancer treatment was not available for all participants, we addressed this limitation in several ways. In the cohort of Medicare-eligible survivors, we conducted sensitivity analysis unadjusted and adjusted for treatment data, and the results were consistent between them as well as with the main results. Furthermore, our analyses were adjusted for stage, which in the absence of treatment data and when treatment is highly standardized and correlated with stage, stage may be a reasonable proxy for broad classifications of treatment.^35^ Moreover, prior studies with the ability to account for detailed treatment data found that increased second cancer risk associated with BMI was independent of treatment among breast cancer survivors.^14^ Additionally, despite our efforts to control for confounders, our study is observational and may still have been subject to unmeasured or residual confounding. While this may preclude from establishing a casual effect, most studies that have established an association of excess body weight with cancer risk have been observational studies.^20^ Experimental studies and mechanistic data also support a possible association of excess body weight with cancer risk,^20^ which may also be relevant to the cause of second cancers.

## Conclusions

In this cohort study of older survivors of nonmetastatic cancer, those who had overweight or obesity at the time of their first cancer diagnosis were at higher risk of developing a second cancer, especially obesity-related cancers. Given the high prevalence of overweight and obesity among cancer survivors, these findings have important public health implications and may inform evidence-based survivorship guidelines to reduce the risk of second primary cancers among cancer survivors. Weight loss strategies and efforts to increase awareness of second cancers among physicians of cancer survivors should be considered.

## Notes

Supplement 1. **eFigure. **Flow Diagram With Inclusion Criteria in the Study **eTable 1.** Definition Codes for Body Mass Index Related Cancers **eTable 2.** Characteristics of the 3,749 Participants in the Cancer Prevention Study II Diagnosed With a Second Primary Cancer **eTable 3.** Risk of Second Cancers With BMI Ascertained at Least 2-years Prior to the First Cancer Diagnosis **eTable 4.** Additionally Adjusted Associations Between Body Mass Index (BMI) and the Risk of Developing a Second Primary Cancers Among 26,894 Participants in the Cancer Prevention Study II Diagnosed With a First Primary Cancer **eTable 5.** Associations Between Body Mass Index (BMI) and the Risk of Developing a Second Primary Cancers Among 26,894 Participants in the Cancer Prevention Study II Diagnosed With a First Primary Cancer by Age **eTable 6.** Associations Between Body Mass Index (BMI) and the Risk of Developing a Second Primary Cancers Among 26,894 Participants in the Cancer Prevention Study II Diagnosed With a First Primary Cancer by Gender **eTable 7.** Associations Between Body Mass Index (BMI) and the Risk of Developing a Second Primary Cancers Among 10,572 Participants in the Cancer Prevention Study II Diagnosed With a First Primary Cancer Among Non-Smokers **eTable 8.** Associations Between Body Mass Index (BMI) and the Risk of Developing a Second Primary Cancers Among 9,935 Participants in the Cancer Prevention Study II Diagnosed With a First Primary Cancer and Physical Activity Information Prior to the First Diagnosis **eTable 9.** Associations Between Body Mass Index (BMI) and the Risk of Developing a Second Primary Cancers Among 11,528 Participants in the Cancer Prevention Study II Diagnosed With a First Primary Cancer Among Medicare-Eligible Only **eTable 10.** Additionally Adjusted Associations Between Body Mass Index (BMI) and the Risk of Developing a Second Primary Cancers Among 11,528 Participants in the Cancer Prevention Study II Diagnosed With a First Primary Cancer Among Medicare-Eligible Only **eTable 11.** Associations Between Body Mass Index (BMI) and the Risk of Developing a Second Primary Cancers Among 10,660 Participants in the Cancer Prevention Study II Diagnosed With a BMI-Related First Primary Cancer **eTable 12.** Associations Between Body Mass Index (BMI) and the Risk of Developing a Second Primary Cancers Among 19,193 Participants in the Cancer Prevention Study II Diagnosed With Any First Cancer Except Breast and Colorectal Cancer **eTable 13.** Associations Between Body Mass Index (BMI) and the Risk of Developing a Second Primary Cancers Among 26,894 Participants in the Cancer Prevention Study II Diagnosed With a First Primary Cancer Using Fine and Gray Regression Models to Account for Competing Risk

Supplement 2.
Data Sharing Statement
