# Supplementary material for: Excess Body Weight and the Risk of Second Primary Cancers Among Cancer Survivors
Source: JAMA Netw Open. 2024 Sep 17;7(9):e2433132. doi: 10.1001/jamanetworkopen.2024.33132 (PMC11409156; doi:10.1001/jamanetworkopen.2024.33132)
Supplement: Supplement 1. — eFigure. Flow Diagram With Inclusion Criteria in the Study eTable 1. Definition Codes for Body Mass Index Related Cancers eTable 2. Characteristics of the 3,749 Participants in the Cancer Prevention Study II Diagnosed With a Second Primary Cancer eTable 3. Risk of Second Cancers With BMI Ascertained at Least 2-years Prior to the First Cancer Diagnosis eTable 4. Additionally Adjusted Associations Between Body Mass Index (BMI) and the Risk of Developing a Second Primary Cancers Among 26,894 Participants in the Cancer Prevention Study II Diagnosed With a First Primary Cancer eTable 5. Associations Between Body Mass Index (BMI) and the Risk of Developing a Second Primary Cancers Among 26,894 Participants in the Cancer Prevention Study II Diagnosed With a First Primary Cancer by Age eTable 6. Associations Between Body Mass Index (BMI) and the Risk of Developing a Second Primary Cancers Among 26,894 Participants in the Cancer Prevention Study II Diagnosed With a First Primary Cancer by Gender eTable 7. Associations Between Body Mass Index (BMI) and the Risk of Developing a Second Primary Cancers Among 10,572 Participants in the Cancer Prevention Study II Diagnosed With a First Primary Cancer Among Non-Smokers eTable 8. Associations Between Body Mass Index (BMI) and the Risk of Developing a Second Primary Cancers Among 9,935 Participants in the Cancer Prevention Study II Diagnosed With a First Primary Cancer and Physical Activity Information Prior to the First Diagnosis eTable 9. Associations Between Body Mass Index (BMI) and the Risk of Developing a Second Primary Cancers Among 11,528 Participants in the Cancer Prevention Study II Diagnosed With a First Primary Cancer Among Medicare-Eligible Only eTable 10. Additionally Adjusted Associations Between Body Mass Index (BMI) and the Risk of Developing a Second Primary Cancers Among 11,528 Participants in the Cancer Prevention Study II Diagnosed With a First Primary Cancer Among Medicare-Eligible Only eTable 11. Associations B [file jamanetwopen-e2433132-s001.pdf]

Supplemental Online Content

Bodelon C, Sung H, Mitchell EL, et al. Excess body weight and the risk of second primary cancers among cancer survivors. *JAMA Netw. Open*. 2024;7(9):e2433132. doi:10.1001/jamanetworkopen.2024.33132

eFigure. Flow Diagram With Inclusion Criteria in the Study
eTable 1. Definition Codes for Body Mass Index Related Cancers
eTable 2. Characteristics of the 3,749 Participants in the Cancer Prevention Study II Diagnosed With a Second Primary Cancer
eTable 3. Risk of Second Cancers With BMI Ascertained at Least 2-years Prior to the First Cancer Diagnosis
eTable 4. Additionally Adjusted Associations Between Body Mass Index (BMI) and the Risk of Developing a Second Primary Cancers Among 26,894 Participants in the Cancer Prevention Study II Diagnosed With a First Primary Cancer
eTable 5. Associations Between Body Mass Index (BMI) and the Risk of Developing a Second Primary Cancers Among 26,894 Participants in the Cancer Prevention Study II Diagnosed With a First Primary Cancer by Age
eTable 6. Associations Between Body Mass Index (BMI) and the Risk of Developing a Second Primary Cancers Among 26,894 Participants in the Cancer Prevention Study II Diagnosed With a First Primary Cancer by Gender
eTable 7. Associations Between Body Mass Index (BMI) and the Risk of Developing a Second Primary Cancers Among 10,572 Participants in the Cancer Prevention Study II Diagnosed With a First Primary Cancer Among Non-Smokers
eTable 8. Associations Between Body Mass Index (BMI) and the Risk of Developing a Second Primary Cancers Among 9,935 Participants in the Cancer Prevention Study II Diagnosed With a First Primary Cancer and Physical Activity Information Prior to the First Diagnosis
eTable 9. Associations Between Body Mass Index (BMI) and the Risk of Developing a Second Primary Cancers Among 11,528 Participants in the Cancer Prevention Study II Diagnosed With a First Primary Cancer Among Medicare-Eligible Only
eTable 10. Additionally Adjusted Associations Between Body Mass Index (BMI) and the Risk of Developing a Second Primary Cancers Among 11,528 Participants in the Cancer Prevention Study II Diagnosed With a First Primary Cancer Among Medicare-Eligible Only
eTable 11. Associations Between Body Mass Index (BMI) and the Risk of Developing a Second Primary Cancers Among 10,660 Participants in the Cancer Prevention Study II Diagnosed With a BMI-Related First Primary Cancer
eTable 12. Associations Between Body Mass Index (BMI) and the Risk of Developing a Second Primary Cancers Among 19,193 Participants in the Cancer Prevention Study II Diagnosed With Any First Cancer Except Breast and Colorectal Cancer
eTable 13. Associations Between Body Mass Index (BMI) and the Risk of Developing a Second Primary Cancers Among 26,894 Participants in the Cancer Prevention Study II Diagnosed With a First Primary Cancer Using Fine and Gray Regression Models to Account for Competing Risk

This supplemental material has been provided by the authors to give readers additional information about their work.

© 2024 Bodelon C et al. *JAMA Network Open*.

eFigure. Flow Diagram With Inclusion Criteria in the Study

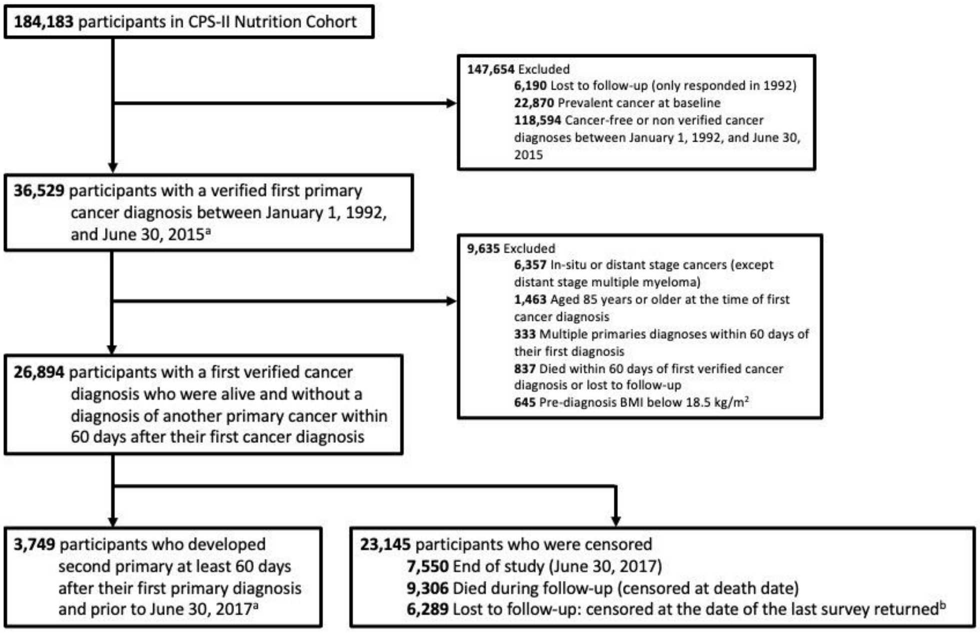

[a]Verified cancers did not include non-melanoma skin cancers.
[b]Lost to follow-up according to last survey return: 271 (4.3%) censored to 1997 survey date; 315 (5.0%) censored to 1999 survey date; 388 (6.2%) censored to 2001 survey date; 435 (6.9%) censored to 2003 survey date; 511 (8.1%) censored to 2005 survey date; 652 (10.4%) censored to 2007 survey date; 791 (12.6%) censored to 2009 survey date; 1,002 (15.9%) censored to 2011 survey date; 811 (12.9%) censored to 2013 survey date; 1,113 (17.7%) censored to 2015 survey date. Follow-up through June 30, 2017, was completed for 88.4% of person-years.

© 2024 Bodelon C et al. *JAMA Network Open*.

eTable 1. Definition Codes for Body Mass Index Related Cancers

Cancer Site	ICD-O-3 codes	Death Codes[a] (ICD-9 and ICD-10 codes)	Histologies to include/exclude
Esophagus adenocarcinoma	C15	150-150.9, C15-C15.9	Only include 8140-8567
Gastric cardia	C16.0	151.0, C16.0	Exclude ≥9590 (blood cancers)
Colon and rectum	C18, C19, C20, Excludes C18.1 (18.1 is appendix)	153-154.9; C18-C20.9; Excludes 153.5, C18.1	Exclude ≥9590 (blood cancers), Exclude non-adenocarcinoma: 8013, 8041, 8070, 8240, 8241, 8244, 8245, 8246, 8720, 8890, 8936, 9540, 8012, 8083, 8120, 8310, 8771, 8850, 8510
Liver	C22.0	155.0, C22.0	Exclude ≥9590 (blood cancers)
Gallbladder	C23	156.0, C23	Exclude ≥9590 (blood cancers)
Pancreas	C25	157-157.9, C25-C25.9	Exclude ≥9590 (blood cancers)
Female Breast	C50	174-1759; C50-C509	Exclude ≥9590 (blood cancers)
Endometrial- corpus uteri	C54, C55.9	179-179.9, 182-182.9, C54-C55.9	Exclude ≥9590 (blood cancers)
Ovary	C56	183-183.9, C56-C56.9	Exclude ≥9590 (blood cancers)
Kidney- renal cell carcinoma	C64.9	189-189.9, C64-C64.9	Only include 8312, 8316, 8317, 8323, 8310, 8319, 8311, 8480, 8260, 8510
Meningioma	C70	192.1, 192.3, C70-C70.9	Only include 9530-9539
Thyroid	C73	193-193.9, C73-C73.9	Exclude ≥9590 (blood cancers)
Multiple Myeloma	--	203-203.9, C90-C90.9	Only include 9732, 9733, 9830

ICD-O: International Classification of Diseases for Oncology; ICD: International Classification of Diseases.
[a]Primary cause of death only.

© 2024 Bodelon C et al. *JAMA Network Open*.

eTable 2. Characteristics of the 3,749 Participants in the Cancer Prevention Study II Diagnosed With a Second Primary Cancer

Characteristics	Survivors, No. (%)			
	All survivors	Survivors by BMI categories		
		18.5≤ BMI <25	25≤ BMI <30	BMI ≥ 30
	(N=3,749)	(N=1,352)	(N=1,719)	(N=678)
Age at second diagnosis, mean (sd)	77.5 (6.3)	78.0 (6.3)	77.6 (6.2)	76.3 (6.3)
Age at second diagnosis				
<65	110 (2.9)	43 (3.2)	40 (2.3)	27 (4.0)
65 - <70	344 (9.2)	106 (7.8)	156 (9.1)	82 (12.1)
70 - <75	757 (20.2)	247 (18.3)	351 (20.4)	159 (23.5)
75 - <80	1,184 (31.6)	414 (30.6)	543 (31.6)	227 (33.5)
80-<85	936 (25.0)	370 (27.4)	437 (25.4)	129 (19.0)
85+	418 (11.1)	172 (12.7)	192 (11.2)	54 (8.0)
Sex				
Female	1,245 (33.2)	551 (40.8)	430 (25.0)	264 (38.9)
Male	2,504 (66.8)	801 (59.2)	1,289 (75.0)	414 (61.1)
Year of second diagnosis				
<2000	578 (15.4)	210 (15.5)	267 (15.5)	101 (14.9)
2001-2005	953 (25.4)	342 (25.3)	436 (25.4)	175 (25.8)
2006-2010	1,134 (30.2)	425 (31.4)	499 (29.0)	210 (31.0)
2011-2017	1,084 (28.9)	375 (27.7)	517 (30.1)	192 (28.3)
Stage of second cancer				
In situ	402 (10.7)	154 (11.4)	189 (11.0)	59 (8.7)
Localized	1,539 (41.1)	548 (40.5)	695 (40.4)	296 (43.7)
Regional	654 (17.4)	229 (16.9)	307 (17.9)	118 (17.4)
Distant	867 (23.1)	328 (24.3)	388 (22.6)	151 (22.3)
Unknown	287 (7.7)	93 (6.9)	140 (8.1)	54 (8.0)
BMI second cancer				
No	2,506 (66.8)	937 (69.3)	1,166 (67.8)	403 (59.4)
Yes	1,243 (33.2)	415 (30.7)	553 (32.2)	275 (40.6)
**Characteristics**	**All survivors**	**Survivors by BMI categories**		
		**18.5≤ BMI <25**	**25≤ BMI <30**	**BMI ≥ 30**

© 2024 Bodelon C et al. *JAMA Network Open*.

Most common second cancers[a]				
Bladder	359 (9.6)	134 (9.9)	171 (9.9)	54 (8.0)
Breast	254 (6.8)	103 (7.6)	92 (5.4)	59 (8.7)
Colorectal	412 (11.0)	113 (8.4)	219 (12.7)	80 (11.8)
Lung	482 (12.9)	184 (13.6)	207 (12.0)	91 (13.4)
Melanoma	344 (9.2)	146 (10.8)	155 (9.0)	43 (6.3)
Non-Hodgkin Lymphoma	379 (10.1)	147 (10.9)	167 (9.7)	65 (9.6)
Prostate	369 (9.8)	118 (8.7)	188 (10.9)	63 (9.3)

[a]Only shown those with frequencies of second cancers greater than 5%.

© 2024 Bodelon C et al. *JAMA Network Open*.

eTable 3. Risk of Second Cancers With BMI Ascertained at Least 2-years Prior to the First Cancer Diagnosis

	All survivors (N=23,747)		1-year survivors (N=21,693)		5-year survivors (N=15,208)	
**BMI variable**	**Num. of 2nd cancers**	**HR (95% CI)[a]**	**Num. of 2nd cancers**	**HR (95% CI)[a]**	**Num. of 2nd cancers**	**HR (95% CI)[a]**
**Outcome: All second cancers**						
Continuous (per 5 Kg/m^2)	3199	1.12 (1.07-1.17)	2842	1.10 (1.05-1.16)	1619	1.09 (1.01-1.16)
Categories						
18.5-24.9	1205	1.00 (Ref.)	1075	1.00 (Ref.)	626	1.00 (Ref.)
25-29.9	1472	1.13 (1.04-1.23)	1314	1.14 (1.05-1.25)	755	1.15 (1.02-1.30)
≥ 30	522	1.31 (1.17-1.47)	453	1.29 (1.15-1.46)	238	1.27 (1.08-1.50)
**Outcome: BMI-related second cancers**						
Continuous (per 5 Kg/m^2)	1032	1.27 (1.18-1.37)	918	1.25 (1.16-1.36)	508	1.25 (1.12-1.40)
Categories						
18.5-24.9	372	1.00 (Ref.)	331	1.00 (Ref.)	177	1.00 (Ref.)
25-29.9	452	1.33 (1.14-1.55)	411	1.36 (1.16-1.61)	240	1.56 (1.25-1.94)
≥ 30	208	1.78 (1.48-2.15)	176	1.70 (1.39-2.09)	91	1.78 (1.34-2.35)

[a]Adjusted for race, smoking (never, former, current, missing) and baseline hazard stratified by age, sex, year of diagnosis, and stage.

Note: BMI obtained from the 1992 survey and restricted to survivors whose 1992 BMI was at least 2 years prior to their first primary diagnosis: median time from 1992 BMI to diagnosis: 8.01 years, IRQ: 4.10-12.76 years.

© 2024 Bodelon C et al. *JAMA Network Open*.

eTable 4. Additionally Adjusted Associations Between Body Mass Index (BMI) and the Risk of Developing a Second Primary Cancers Among 26,894 Participants in the Cancer Prevention Study II Diagnosed With a First Primary Cancer

	**All survivors (N=26,894)**		**1-year survivors (N=24,580)**		**5-year survivors (N=17,473)**	
**BMI variable**	**Num. of 2nd cancers**	**HR (95% CI)[a]**	**Num. of 2nd cancers**	**HR (95% CI)[a]**	**Num. of 2nd cancers**	**HR (95% CI)[a]**
**Outcome: All second cancers**						
Continuous (per 5 Kg/m^2)	3749	1.11 (1.06-1.16)	3,356	1.10 (1.05-1.15)	1,979	1.10 (1.03-1.17)
Categories						
18.5-24.9	1352	1.00 (Ref.)	1,217	1.00 (Ref.)	736	1.00 (Ref.)
25-29.9	1719	1.13 (1.04-1.22)	1,546	1.14 (1.05-1.24)	908	1.11 (1.00-1.24)
≥ 30	678	1.29 (1.16-1.43)	593	1.26 (1.13-1.41)	335	1.26 (1.09-1.46)
**Outcome: BMI-related second cancers**						
Continuous (per 5 Kg/m^2)	1243	1.25 (1.17-1.34)	1,117	1.25 (1.16-1.34)	638	1.25 (1.14-1.38)
Categories						
18.5-24.9	415	1.00 (Ref.)	373	1.00 (Ref.)	214	1.00 (Ref.)
25-29.9	553	1.37 (1.19-1.59)	502	1.39 (1.20-1.62)	292	1.42 (1.16-1.73)
≥ 30	275	1.69 (1.42-2.02)	242	1.65 (1.37-1.99)	132	1.64 (1.28-2.10)

[a]Adjusted for race, smoking, surgery (no, yes, unknown), chemotherapy (no, yes, unknown), radiation therapy (no, yes, unknown), diabetes (no, yes), hypertension (no, yes) and prevalent cardiovascular disease (no, yes) and baseline hazard stratified by age, sex, year of diagnosis, and stage.

© 2024 Bodelon C et al. *JAMA Network Open*.

eTable 5. Associations Between Body Mass Index (BMI) and the Risk of Developing a Second Primary Cancers Among 26,894 Participants in the Cancer Prevention Study II Diagnosed With a First Primary Cancer by Age

	**All survivors < 70 years old (N=9,842)**		**All survivors ≥ 70 years old (N=17,052)**	
**BMI variable**	**Num. of 2nd cancers**	**HR (95% CI)[a]**	**Num. of 2nd cancers**	**HR (95% CI)[a]**
**Outcome: All second cancers**				
Continuous (per 5 Kg/m^2)	1,599	1.16 (1.09-1.24)	2,150	1.10 (1.04-1.17)
Categories				
18.5-24.9	541	1.00 (Ref.)	811	1.00 (Ref.)
25-29.9	732	1.20 (1.06-1.36)	987	1.12 (1.01-1.24)
≥ 30	326	1.46 (1.26-1.70)	352	1.25 (1.09-1.43)
**Outcome: BMI-related second cancers**				
Continuous (per 5 Kg/m^2)	555	1.32 (1.20-1.46)	688	1.25 (1.15-1.37)
Categories				
18.5-24.9	174	1.00 (Ref.)	241	1.00 (Ref.)
25-29.9	241	1.47 (1.18-1.83)	312	1.35 (1.13-1.63)
≥ 30	140	2.09 (1.63-2.68)	135	1.56 (1.24-1.96)

[a]Adjusted for race, smoking and baseline hazard stratified by age, sex, year of diagnosis, and stage.

© 2024 Bodelon C et al. *JAMA Network Open*.

eTable 6. Associations Between Body Mass Index (BMI) and the Risk of Developing a Second Primary Cancers Among 26,894 Participants in the Cancer Prevention Study II Diagnosed With a First Primary Cancer by Gender

	All female survivors (N=10,974)		All male survivors (N=15,920)	
**BMI variable**	**Num. of 2nd cancers**	**HR (95% CI)[a]**	**Num. of 2nd cancers**	**HR (95% CI)[a]**
**Outcome: All second cancers**				
Continuous (per 5 Kg/m^2)	1,245	1.09 (1.03-1.16)	2,504	1.16 (1.09-1.23)
Categories				
18.5-24.9	551	1.00 (Ref.)	801	1.00 (Ref.)
25-29.9	430	1.18 (1.02-1.36)	1,289	1.14 (1.03-1.25)
≥ 30	264	1.28 (1.09-1.51)	414	1.36 (1.20-1.55)
**Outcome: BMI-related second cancers**				
Continuous (per 5 Kg/m^2)	665	1.23 (1.13-1.33)	578	1.37 (1.23-1.54)
Categories				
18.5-24.9	257	1.00 (Ref.)	158	1.00 (Ref.)
25-29.9	245	1.37 (1.13-1.67)	308	1.41 (1.15-1.74)
≥ 30	163	1.68 (1.35-2.09)	112	1.90 (1.46-2.48)

[a]Adjusted for race, smoking and baseline hazard stratified by age, year of diagnosis, and stage.

© 2024 Bodelon C et al. *JAMA Network Open*.

eTable 7. Associations Between Body Mass Index (BMI) and the Risk of Developing a Second Primary Cancers Among 10,572 Participants in the Cancer Prevention Study II Diagnosed With a First Primary Cancer Among Non-Smokers

	All survivors (N=10,572)		1-year survivors (N=9,867)		5-year survivors (N=7,337)	
**BMI variable**	**Num. of 2nd cancers**	**HR (95% CI)[a]**	**Num. of 2nd cancers**	**HR (95% CI)[*]**	**Num. of 2nd cancers**	**HR (95% CI)[a]**
**Outcome: All second cancers**						
Continuous (per 5 Kg/m^2)	1288	1.09 (1.01-1.18)	1172	1.09 (1.01-1.18)	722	1.10 (0.99-1.22)
Categories						
18.5-24.9	502	1.00 (Ref.)	459	1.00 (Ref.)	285	1.00 (Ref.)
25-29.9	548	1.14 (0.99-1.31)	497	1.12 (0.96-1.30)	316	1.16 (0.95-1.40)
≥ 30	238	1.28 (1.07-1.54)	216	1.26 (1.04-1.53)	121	1.18 (0.91-1.52)
**Outcome: BMI-related second cancers**						
Continuous (per 5 Kg/m^2)	506	1.25 (1.12-1.39)	466	1.26 (1.12-1.41)	279	1.36 (1.17-1.59)
Categories						
18.5-24.9	182	1.00 (Ref.)	165	1.00 (Ref.)	101	1.00 (Ref.)
25-29.9	209	1.34 (1.06-1.70)	195	1.40 (1.09-1.79)	119	1.41 (1.02-1.94)
≥ 30	115	1.63 (1.24-2.16)	106	1.64 (1.23-2.20)	59	1.70 (1.15-2.50)

[a]Adjusted for race, and baseline hazard stratified by age, sex, year of diagnosis, and stage.

© 2024 Bodelon C et al. *JAMA Network Open*.

eTable 8. Associations Between Body Mass Index (BMI) and the Risk of Developing a Second Primary Cancers Among 9,935 Participants in the Cancer Prevention Study II Diagnosed With a First Primary Cancer and Physical Activity Information Prior to the First Diagnosis[a]

	**All survivors (N=9,935)**		**1-year survivors (N=9,057)**		**5-year survivors (N=6,300)**	
**BMI variable**	**Num. of 2nd cancers**	**HR (95% CI)[b]**	**Num. of 2nd cancers**	**HR (95% CI)[b]**	**Num. of 2nd cancers**	**HR (95% CI)[b]**
**Outcome: All second cancers**						
Continuous (per 5 Kg/m^2)	1231	1.09 (1.01-1.17)	1081	1.08 (1.00-1.17)	534	1.05 (0.93-1.18)
Categories						
18.5-24.9	432	1.00 (REF)	380	1.00 (REF)	193	1.00 (REF)
25-29.9	563	1.12 (0.97-1.29)	497	1.14 (0.98-1.32)	258	1.16 (0.94-1.43)
≥ 30	236	1.28 (1.07-1.54)	204	1.25 (1.03-1.52)	83	1.06 (0.79-1.42)
**Outcome: BMI-related second cancers**						
Continuous (per 5 Kg/m^2)	418	1.23 (1.09-1.38)	373	1.23 (1.08-1.40)	189	1.24 (1.03-1.48)
Categories						
18.5-24.9	128	1.00 (REF)	112	1.00 (REF)	59	1.00 (REF)
25-29.9	196	1.54 (1.19-1.99)	180	1.62 (1.23-2.13)	94	1.62 (1.12-2.36)
≥ 30	94	1.73 (1.27-2.36)	81	1.69 (1.21-2.36)	36	1.61 (1.00-2.60)

[a]Only available in the 1999, 2001, 2005, 2009 and 2011 surveys. This cohort was made up of survivors with pre-diagnosis BMI ascertained from the above surveys and have non-missing physical activity data in that survey.

[b]Adjusted for race, smoking and physical activity (<7.5, 7.5 - <15, and 15+ MET-hours/week) and baseline hazard stratified by age, sex, year of diagnosis, and stage. MET: metabolic equivalent.

© 2024 Bodelon C et al. *JAMA Network Open*.

eTable 9. Associations Between Body Mass Index (BMI) and the Risk of Developing a Second Primary Cancers Among 11,528 Participants in the Cancer Prevention Study II Diagnosed With a First Primary Cancer Among Medicare-Eligible Only

	**All survivors (N=11,528)**		**1-year survivors (N=10,461)**		**5-year survivors (N=7,045)**	
**BMI variable**	**Num. of 2nd cancers**	**HR (95% CI)***	**Num. of 2nd cancers**	**HR (95% CI)***	**Num. of 2nd cancers**	**HR (95% CI)***
**Outcome: All second cancers**						
Continuous (per 5 Kg/m^2)	1427	1.12 (1.05-1.20)	1239	1.11 (1.03-1.20)	601	1.09 (0.98-1.22)
Categories						
18.5-24.9	500	1.00 (Ref.)	434	1.00 (Ref.)	215	1.00 (Ref.)
25-29.9	665	1.20 (1.06-1.37)	588	1.22 (1.06-1.40)	292	1.26 (1.03-1.53)
≥ 30	262	1.32 (1.11-1.56)	217	1.25 (1.04-1.50)	94	1.17 (0.89-1.53)
**Outcome: BMI-related second cancers**						
Continuous (per 5 Kg/m^2)	475	1.29 (1.16-1.44)	422	1.28 (1.14-1.44)	201	1.26 (1.06-1.49)
Categories						
18.5-24.9	146	1.00 (Ref.)	131	1.00 (Ref.)	63	1.00 (Ref.)
25-29.9	224	1.54 (1.22-1.94)	203	1.59 (1.24-2.03)	101	1.61 (1.13-2.29)
≥ 30	105	1.81 (1.36-2.39)	88	1.69 (1.25-2.29)	37	1.56 (0.99-2.44)

*Adjusted for race, smoking and baseline hazard stratified by age, sex, year of diagnosis, and stage.

© 2024 Bodelon C et al. *JAMA Network Open*.

eTable 10. Additionally Adjusted Associations Between Body Mass Index (BMI) and the Risk of Developing a Second Primary Cancers Among 11,528 Participants in the Cancer Prevention Study II Diagnosed With a First Primary Cancer Among Medicare-Eligible Only

	All survivors (N=11,528)		1-year survivors (N=10,461)		5-year survivors (N=7,045)	
**BMI variable**	**Num. of 2nd cancers**	**HR (95% CI)***	**Num. of 2nd cancers**	**HR (95% CI)***	**Num. of 2nd cancers**	**HR (95% CI)***
**Outcome: All second cancers**						
Continuous (per 5 Kg/m^2)	1,427	1.11 (1.03-1.19)	1,239	1.10 (1.02-1.19)	601	1.08 (0.96-1.21)
Categories						
18.5-24.9	500	1.00 (Ref.)	434	1.00 (Ref.)	215	1.00 (Ref.)
25-29.9	665	1.17 (1.03-1.34)	588	1.20 (1.04-1.38)	292	1.24 (1.01-1.51)
≥ 30	262	1.27 (1.07-1.51)	217	1.22 (1.01-1.47)	94	1.14 (0.86-1.51)
**Outcome: BMI-related second cancers**						
Continuous (per 5 Kg/m^2)	475	1.29 (1.15-1.44)	422	1.28 (1.13-1.44)	201	1.22 (1.02-1.46)
Categories						
18.5-24.9	146	1.00 (Ref.)	131	1.00 (Ref.)	63	1.00 (Ref.)
25-29.9	224	1.53 (1.21-1.94)	203	1.58 (1.23-2.03)	101	1.56 (1.09-2.24)
≥ 30	105	1.77 (1.32-2.38)	88	1.64 (1.20-2.26)	37	1.46 (0.92-2.33)

*Adjusted for race, smoking, surgery (no, yes, unknown), chemotherapy (no, yes, unknown), radiation therapy (no, yes, unknown), diabetes (no, yes), hypertension (no, yes) and prevalent cardiovascular disease (no, yes) and baseline hazard stratified by age, sex, year of diagnosis, and stage.

© 2024 Bodelon C et al. *JAMA Network Open*.

eTable 11. Associations Between Body Mass Index (BMI) and the Risk of Developing a Second Primary Cancers Among 10,660 Participants in the Cancer Prevention Study II Diagnosed With a BMI-Related First Primary Cancer

	All survivors (N=10,660)		1-year survivors (N=9,905)		5-year survivors (N=6,914)	
**BMI variable**	**Num. of 2nd cancers**	**HR (95% CI)***	**Num. of 2nd cancers**	**HR (95% CI)***	**Num. of 2nd cancers**	**HR (95% CI)***
**Outcome: All second cancers**						
Continuous (per 5 Kg/m^2)	1292	1.09 (1.01-1.16)	1169	1.08 (1.01-1.17)	698	1.03 (0.93-1.13)
Categories						
18.5-24.9	516	1.00 (Ref.)	469	1.00 (Ref.)	302	1.00 (Ref.)
25-29.9	502	1.18 (1.02-1.37)	455	1.19 (1.02-1.39)	262	1.12 (0.91-1.37)
≥ 30	274	1.23 (1.03-1.47)	245	1.23 (1.02-1.48)	134	1.05 (0.81-1.35)
**Outcome: BMI-related second cancers**						
Continuous (per 5 Kg/m^2)	524	1.27 (1.15-1.39)	482	1.27 (1.15-1.40)	270	1.21 (1.06-1.38)
Categories						
18.5-24.9	183	1.00 (Ref.)	168	1.00 (Ref.)	104	1.00 (Ref.)
25-29.9	208	1.56 (1.24-1.96)	192	1.56 (1.23-1.98)	107	1.54 (1.13-2.10)
≥ 30	133	1.73 (1.34-2.24)	122	1.75 (1.34-2.30)	59	1.44 (1.00-2.08)

*Adjusted for race, smoking and baseline hazard stratified by age, sex, year of diagnosis, and stage.

© 2024 Bodelon C et al. *JAMA Network Open*.

eTable 12. Associations Between Body Mass Index (BMI) and the Risk of Developing a Second Primary Cancers Among 19,193 Participants in the Cancer Prevention Study II Diagnosed With Any First Cancer Except Breast and Colorectal Cancer

	**All survivors (N=19,193)**		**1-year survivors (N=17,190)**		**5-year survivors (N=12,004)**	
**BMI variable**	**Num. of 2nd cancers**	**HR (95% CI)***	**Num. of 2nd cancers**	**HR (95% CI)***	**Num. of 2nd cancers**	**HR (95% CI)***
**Outcome: All second cancers**						
Continuous (per 5 Kg/m^2)	2786	1.15 (1.09-1.22)	2476	1.14 (1.07-1.20)	1439	1.15 (1.07-1.24)
Categories						
18.5-24.9	951	1.00 (Ref.)	850	1.00 (Ref.)	494	1.00 (Ref.)
25-29.9	1338	1.16 (1.06-1.28)	1198	1.18 (1.07-1.30)	710	1.17 (1.03-1.33)
≥ 30	497	1.40 (1.24-1.59)	428	1.35 (1.19-1.55)	235	1.36 (1.14-1.63)
**Outcome: BMI-related second cancers**						
Continuous (per 5 Kg/m^2)	881	1.31 (1.20-1.43)	778	1.28 (1.17-1.41)	443	1.35 (1.19-1.54)
Categories						
18.5-24.9	284	1.00 (Ref.)	250	1.00 (Ref.)	135	1.00 (Ref.)
25-29.9	402	1.40 (1.17-1.68)	364	1.47 (1.21-1.78)	217	1.49 (1.16-1.93)
≥ 30	195	1.93 (1.56-2.40)	164	1.80 (1.42-2.28)	91	1.90 (1.38-2.61)

*Adjusted for race, smoking and baseline hazard stratified by age, sex, year of diagnosis, and stage.

© 2024 Bodelon C et al. *JAMA Network Open*.

eTable 13. Associations Between Body Mass Index (BMI) and the Risk of Developing a Second Primary Cancers Among 26,894 Participants in the Cancer Prevention Study II Diagnosed With a First Primary Cancer Using Fine and Gray Regression Models to Account for Competing Risk

	**All survivors (N=26,894)**		**1-year survivors (N=24,580)**		**5-year survivors (N=17,473)**	
**BMI variable**	**Num. of 2nd cancers**	**HR (95% CI)***	**Num. of 2nd cancers**	**HR (95% CI)***	**Num. of 2nd cancers**	**HR (95% CI)***
**Outcome: All second cancers**						
Continuous (per 5 Kg/m^2)	3749	1.10 (1.06-1.15)	3356	1.09 (1.05-1.14)	1979	1.08 (1.02-1.14)
Categories						
18.5-24.9	1352	1.00 (Ref.)	1217	1.00 (Ref.)	736	1.00 (Ref.)
25-29.9	1719	1.15 (1.07-1.24)	1546	1.15 (1.06-1.24)	908	1.11 (1.00-1.23)
≥ 30	678	1.28 (1.16-1.41)	593	1.24 (1.12-1.38)	335	1.20 (1.05-1.38)
**Outcome: BMI-related second cancers**						
Continuous (per 5 Kg/m^2)	1243	1.25 (1.17-1.32)	1117	1.24 (1.16-1.32)	638	1.23 (1.13-1.34)
Categories						
18.5-24.9	415	1.00 (Ref.)	373	1.00 (Ref.)	214	1.00 (Ref.)
25-29.9	553	1.37 (1.20-1.56)	502	1.38 (1.20-1.58)	292	1.39 (1.16-1.68)
≥ 30	275	1.70 (1.46-2.00)	242	1.65 (1.40-1.95)	132	1.59 (1.27-2.00)

*Adjusted for race, smoking and baseline hazard stratified by age, sex, year of diagnosis, and stage.

© 2024 Bodelon C et al. *JAMA Network Open*.
