# Supplementary material for: Excess Body Weight and the Risk of Second Primary Cancers Among Cancer Survivors
Source: JAMA Netw Open. 2024 Sep 17;7(9):e2433132. doi: 10.1001/jamanetworkopen.2024.33132 (PMC11409156; doi:10.1001/jamanetworkopen.2024.33132)
Supplement: Supplement 2. — Data Sharing Statement [file jamanetwopen-e2433132-s002.pdf]

Data Sharing Statement

Bodelon. Excess Body Weight and the Risk of Second Primary Cancers Among Cancer Survivors. *JAMA Netw Open*. Published September 17, 2024. doi:10.1001/jamanetworkopen.2024.33132

Data
Data available: Yes
Data types: Deidentified participant data
How to access data: Data are available from the American Cancer Society (ACS) by following the ACS Data Access Procedures (https://www.cancer.org/content/dam/cancer-org/research/epidemiology/cancer-prevention-study-data-access-policies.pdf) for researchers who meet the criteria for access to confidential data. Please email cohort.data@cancer.org to inquire about access.
When available: With publication

Supporting Documents
Document types: None

Additional Information
Who can access the data: Researchers after an approved proposal
Types of analyses: Analytical dataset
Mechanisms of data availability: with investigator support
